# A noval pulmonary function evaluation method based on ResNet50 + SVR model and cough

**DOI:** 10.1038/s41598-023-49334-4

**Published:** 2023-12-12

**Authors:** Wenlong Xu, Guoqiang He, Dan Shen, Bingqiao Xu, Peirong Jiang, Feng Liu, Xiaomin Lou, Lingling Guo, Li Ma

**Affiliations:** 1 College of Information Engineering, China Jiliang University, Hangzhou, China; 2 The First Affiliated Hospital, College of Medicine, Zhejiang University, Hangzhou, China; 3 Lishui People’s Hospital, Lishui, China; 4 School of Information Technology and Electrical Engineering, University of Queensland, Brisbane, Australia; 5 Hangzhou Chest Hospital Affiliated, Zhejiang University Medical College, Hangzhou, China; 6 College of Chemical Engineering, Zhejiang University of Technology, Hangzhou, China; 7 Zhejiang Provincial People’s Hospital, Affiliated People’s Hospital, Hangzhou Medical College, Hangzhou, China

**Keywords:** Diseases, Health care

## Abstract

Traditionally, the clinical evaluation of respiratory diseases was pulmonary function testing, which can be used for the detection of severity and prognosis through pulmonary function parameters. However, this method is limited by the complex process, which is impossible for patients to monitor daily. In order to evaluate pulmonary function parameters conveniently with less time and location restrictions, cough sound is the substitute parameter. In this paper, 371 cough sounds segments from 150 individuals were separated into 309 and 62 as the training and test samples. Short-time Fourier transform (STFT) was applied to transform cough sound into spectrogram, and ResNet50 model was used to extract 2048-dimensional features. Through support vector regression (SVR) model with biological attributes, the data were regressed with pulmonary function parameters, FEV1, FEV1%, FEV1/FVC, FVC, FVC%, and the performance of this models was evaluated with fivefold cross-validation. Combines with deep learning and machine learning technologies, the better results in the case of small samples were achieved. Using the coefficient of determination (R^2^), the ResNet50 + SVR model shows best performance in five basic pulmonary function parameters evaluation as FEV1(0.94), FEV1%(0.84), FEV1/FVC(0.68), FVC(0.92), and FVC%(0.72). This ResNet50 + SVR hybrid model shows excellent evaluation of pulmonary function parameters during coughing, making it possible to realize a simple and rapid evaluation for pneumonia patients. The technology implemented in this paper is beneficial in judge the patient's condition, realize early screening of respiratory diseases, evaluate postoperative disease changes and detect respiratory infectious diseases without time and location restrictions.

## Introduction

According to the World Health Organization's worldwide survey in 2021, three of the world's top ten common fatal diseases were respiratory diseases^1^. By 2021, respiratory diseases such as chronic obstructive pulmonary disease (COPD), lung cancer and acute respiratory infections account for approximately 7 million deaths per year^1^. With the increasing aging population and the rising smoking rate in developing countries, the prevalence of respiratory diseases is expected to continue to rise in the next 40 years, with an estimated annual death more than 5.4 million by 2060^2^. Nowadays, asthma and Chronic obstructive pulmonary disease (COPD) are the most common respiratory diseases, affecting more than 500 million people worldwide^3^. The problem of respiratory disease has been widespread in modern society. Early detection and daily monitoring of pulmonary function are the key in disease control and prevention.

Pulmonary function testing (PFT) is the most practical method for clinical diagnosis and research in respiratory diseases^4^, and it is the basic method to assist doctors in diagnosing respiratory diseases^5^. In a typical clinical diagnosis, the pulmonary function testing can feed back whether the tester have respiratory diseases and the type and severity of the disease according to the pulmonary function parameters. During the pulmonary function testing, the tester needs to bite the adapter of the spirometer, inhale hard and exhale quickly to complete the lung ventilation function test. Since only three consistent measurement results can be used, the tester must apply maximum inhalation and exhalation force to complete this test^6^. In the PFT results, five basic pulmonary function parameters are Forced expiratory volume in one second (FEV1), Measured FEV1 value/Reference FEV1 value (FEV1%), Forced vital capacity (FVC), Measured FVC value/Reference FVC value (FVC%) and Forced expiratory volume in one second/Total vital capacity (FEV1/FVC)^7^. In the diagnosis of obstructive respiratory disease^8^, FEV1 of patients decreased significantly; while, the decrease of FVC was negligible, making the FEV1/FVC less than 0.7. In the diagnosis of restrictive respiratory disease^8^, both FEV1 and FVC of patients decreased, and FVC has fallen further than FEV1, resulting in the ratio of FEV1/FVC higher than 0.8. Finally, the severity is divided into mild, moderate, and severe stages through FEV1% and FVC%^9^.

Chronic respiratory diseases patients need to regularly monitor their pulmonary function for a period to prevent the aggravation of the disease and the risk of death. However, high test fee^10^ and complex process^11^ prevent the wide distribution of the PFT. Although it is a mature detection method, PFT is impossible to meet the daily testing of patients with respiratory diseases. PFT requires a pulmonary function instrument to be performed. Currently, only large and medium-sized hospitals in cities have specialized pulmonary function departments, while small hospitals in street communities are unable to afford the purchase and maintenance costs, resulting in patients being unable to perform daily examinations. Moreover, the direct cost of a PFT for patients is also high. In addition, the process of PFT is complex and strict, requiring close coordination and multiple repetitions between doctors and subjects to achieve, which may be inconvenient and difficult to complete for the elderly and patients with severe respiratory diseases. Furthermore, even if patients have already experienced early symptoms of respiratory diseases, such as coughing and poor breathing, they often do not choose to seek medical examination in the first place^12^. The compensatory mechanism of the human body can easily lead to delays in the patient's condition, and patients may think that their own disease is just a common infection caused by influenza and do not take it seriously. In addition, more than 50% of patients^13^ with chronic respiratory diseases live in low—and middle-income countries with limited medical resources and knowledge. Even in large cities with abundant medical resources, overcrowding during specific influenza seasons can bring inconvenience to respiratory disease patients. Therefore, chronic respiratory disease patients urgent need convenient, low-cost, and more availability technologies and methods to estimate their pulmonary function.

In contrast, spontaneous cough data can be easily captured, and smartphones can be used to record and transmit it to the internet. Therefore, it is comfortable, non-invasive, and non-contact methods to estimate the pulmonary function through cough sound, and chronic respiratory disease patients can complete estimation without leaving home or needing professional help. Firstly, the action of cough is similar as the action of pulmonary ventilation function testing. Physiologically, cough and pulmonary ventilation function testing consists of three stages: inhalation, compression, and exhalation^14^, i.e., a certain amount of air is inhaled, and then the maximum chest volume is reached, and the gas is exhaled quickly and forcefully by chest pressure. Secondly, cough is a common symptom of respiratory diseases^15^, which is affected by airflow, the acoustic properties of tissues, and the shape of the lungs and airways. As a protective physiological reflex action, cough reflects the presence of mucus on the surface of the lungs and airways, the amount of mucus, and the ability of the lungs to expand^16^. Since different respiratory diseases exhibit different pathological effects on tissues and organs, and cough sounds reflect different characteristics^17^. Such studies on the relationship between cough sounds and lung function parameters opens the possibilities for new clinical diagnosis and disease control of respiratory diseases.

This study aims to implement a promoted method to estimate comprehensive pulmonary function parameters based on cough sound, which are essential indicators for clinical diagnosis of the type and severity of respiratory diseases. Furthermore, the research subjects include patients with chronic obstructive pulmonary disease, asthma, bronchitis, pulmonary nodules, lung cancer, and varying degrees of obstructive, restrictive, and mixed respiratory diseases. The major contributions of this paper are summarized as follows.

1. We propose a non-contact, non-invasive, and comfortable pulmonary function parameter evaluation method using cough sounds to meet the daily monitoring needs of patients with chronic respiratory diseases.
2. We use multiple deep learning to extract image features and machine learning regression model to evaluate five lung function parameters, which refined the learning process of pulmonary function parameters and improved the evaluation accuracy.

The structure of this paper is organized as follows. In Section "Related work", we present an overview of the related works. In Section "Materials and methods", we introduce the detail process of data acquisition and feature extraction for cough sound images, as well as the implementation of the regression model. In Section "Results", we provided a detail introduction to the experimental results. In Section "Conclusion", we conclude this study.

## Related work

### Pulmonary function evaluation based on sound signal

Sounds have been used in recent research about pulmonary function evaluation. With the rise of medical digitization, the diagnosis and evaluation of respiratory diseases based on sound signals have significant advantages in convenience and cost when compared to traditional pulmonary function testing. Previously, Alam^18^ reported that 323 speech and respiratory sounds were used to estimated FEV1% parameters by support vector machine, linear regression, and random forest model, achieving RMSE = 10.86. According to Salehen’s^19^ study, "a" and "AAAA" sounds from 201 subjects were used to estimated FEV1/FVC parameters by multi-layer perceptron model, obtaining MAE = 6.7. 59 subjects collected from mobile phones were used to estimated FEV1/FVC parameters by neural network regression model, achieving MAE = 8.6 in San Chun’s^20^ work. Moreover, it has been proved that cough sounds can be used to estimated pulmonary function parameters.

### Pulmonary function evaluation based on cough sound

The most common symptom of respiratory disease is coughing, which stimulates secretion in the airway as a protective measure. According to clinical researches, the severity of cough is an important indicator of the occurrence and progression of respiratory diseases. 16 healthy people and 12 patients cough and wheeze were used to evaluate FEV1% and FVC% parameters with support vector regression model, achieving RMSE of 11.06 and 10.3 in Rao’s investigation^21^. Sharan^22^ collected cough sounds of 322 adults to estimate FEV1, FVC, FEV1/FVC parameters with random forest and support vector regression. According to our previous work^23^, cough sounds from 133 participants was extracted and multiple machine learning regression models were applied. The coefficient determination showed that among these models, Support vector regression model had the highest accuracy in evaluating FVC (0.84), FEV1% (0.61), and FVC% (0.62); while, the gradient enhanced regression model showed better results in FEV1 (0.86) and FEV1/FVC (0.54). However, the result is far from pretty using machine learning models mentioned above to evaluate pulmonary function parameters as the manually extracted cough sound features were incomplete, leading to the poor accuracy of subsequent fitting.

Cough is one of the early symptoms of COVID-19, and cough sound can be used to detect COVID-19. According to MIT^24^ an artificial intelligence speech processing framework was developed to screen COVID-19 from 4256 cough sounds in 1064 subjects using a convolutional neural network model using the characteristics of cough acoustic signal processing. The model's sensitivity and specificity to detect COVID-19 was 98.5% and 94.2%, respectively, where the model to detect asymptomatic patients was 100%. Online medical counseling methods based on cough sounds have emerged in recent years. In Nemati^25^ studies, devices such as mobile apps and wearable technologies have demonstrated their feasibility in diagnosing different respiratory diseases. The cough sounds were collected through the mobile app and feedback from the user through the Internet, and to estimate whether the subject had pulmonary obstructive diseases such as COPD and asthma. Kosasih et al.^26^ proposed a method to detect multiple respiratory diseases by analyzing cough sounds and sending sounds to an AI model containing a smartphone. This method uses various classifiers including LR, ANN, SVM, RF to select the best performing classification model, and finally achieves the sensitivity, specificity and accuracy of 86%, 91% and 91%, respectively.

In this work, using the cough sound spectrogram as the model input will greatly improve the feature coverage (time, frequency, and energy), and using the hybrid model of deep learning and machine learning to estimate five pulmonary function parameters can obtain a better accuracy and lower error. In addition, our work added the ratio of FEV1/FVC, which is important indicator for clinical diagnosis of the type and severity of pulmonary function diseases. Furthermore, spectral features were automatically extracted through deep learning technology, avoiding feature selection, and improving fitting accuracy.

## Materials and methods

**Figure 1:**
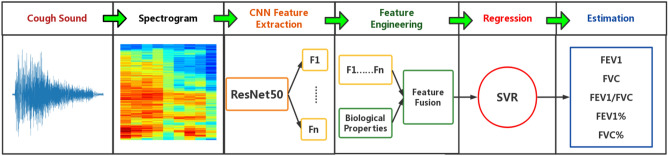
Procedures for estimating pulmonary function parameters.

The procedure for estimating pulmonary function parameters is shown in Fig. 1. Totally, 371 cough sounds segments were separated from 150 patients who underwent pulmonary function testing with software Audacity^27^. Firstly, short-time Fourier transform (STFT) was applied to transform cough sound into spectrogram. Secondly, ResNet50 model is used to extract 2048-dimensional image features from the spectrogram and fused 4-dimensional biological attribute features. Finally, using 2052-dimensional feature of cough sounds to train support vector regression (SVR) model, which can realize the estimation of pulmonary function parameters.

### Data

Cough sounds from 150 subjects and five pulmonary function parameters (i.e., FEV1, FEV1%, FVC, FVC% and FEV1/FVC) were recorded on the advice of doctors. The consent was approved by the Ethics Committee of Lishui People’s Hospital, Lishui, China, (Approval No. [2022] (020-01)). All methods were carried out in accordance with relevant guidelines and regulations and informed consent was obtained from all subjects. Using Audacity^27^, 371 cough sound segments with a length of 350 ms were separated from 150 patients cough sounds. Each cough sound segment was annotated with five pulmonary function parameters and patients’ biological attributes (e.g., sex, age, height, and weight). These 371 cough sound data sets were randomly selected and divided into 309 training sets and 62 test sets. The collection process is as standardized and quiet as possible to prevent related errors. Subjects were recorded at the field by physicians, the smartphone microphone port was placed approximately 40 cm from the subject's mouth at an angle of approximately 45 degrees, and the microphone sampling frequency was 16,000 Hz. Each subject was instructed to cough at least three times in 30 s, with a continuous cough interval of approximately 1 s. Because the cough sounds of pulmonary function subjects belong to special biological information and need more complex privacy protection procedures, in order to prevent sensitive information leakage and maintain the ambiguity of patient personal information, this study only retain biological characteristics of the age, sex, height, weight, cough voice and pulmonary function parameters data, without retaining any private information. Table 1 shows the breakdown of subjects' biological attributes and cough data, and the pulmonary function results were collected under the professional guidelines for pulmonary function testing^28^. Among these 150 subjects, 77, 32, 31, and 10 of them show normal pulmonary function, mild abnormal pulmonary function, moderate abnormal pulmonary function, and severe abnormal pulmonary function, respectively. Among these 371 cough sounds, 201, 81, 69, and 20 of them show normal pulmonary function, mild abnormal pulmonary function, moderate abnormal pulmonary function, and severe abnormal pulmonary function, respectively. The majority of the dataset is adults, with an average age of 60.39 years old, with more male members and 97 participants. Their average height is 162.23 cm, and their average weight is 64.13 kg. The Table 1 shows a breakdown based on pulmonary function.

**Table 1:** Statistical overview of demographic and cough data.

	Normal	Mildly	Moderately	Severely	Total
Number of subjects	77	32	31	10	150
Number of coughs	201	81	69	20	371
Age mean in years	63.84	53.83	67.45	78	60.39
Gender (male: female)	48:29	18:14	23:8	7:1	97:53
Height mean (cm)	162.76	161.69	161.13	163.3	162.23
Weight mean (kg)	63.57	62.84	69.45	56	64.13

### Cough sounds analysis

**Figure 2:**
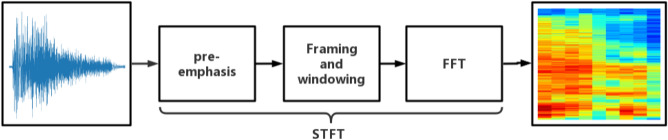
Cough sound transformation spectrogram process.

Since cough sounds contains a variety of respiratory information, it is required to express the information as much as possible. Compared with signal processing technology, spectrogram, a 2-dimensional image, can reflect the dynamic changes of cough sound signal in energy, frequency, and time, which can establish more comprehensive information system. The abscissa in spectrogram is time, the ordinate is frequency, and the coordinate point is the energy with different shades. Further, spectrogram can be constructed by Short-Time Fourier Transform (STFT), and Fig. 2 shows the process. Hamming window is used as window function with length of 1024, the FFT length is 1024, and the overlap length is 512. The spectrogram formula for constructing cough sounds using STFT is:

1 $${\text{F}}({\text{n}},\upomega )=\sum_{{\text{i}}=-\infty }^{\infty }{\text{x}}({\text{i}})\upomega ({\text{n}}-{\text{i}}){e}^{-j\omega n}$$

where ${\text{x}}({\text{i}})$ represents a cough sound; $\upomega ({\text{n}}-{\text{i}})$ represents window functions; and ${|{\text{F}}({\text{n}},\upomega )|}^{2}$ is a spectrum diagram.

### Feature engineering

#### Feature extraction

Convolutional neural network (CNN)^29^ is a commonly used image processing method, which can automatically extract image features to reduce the incompleteness caused by artificial design features. It extracts image features through convolution operation of convolution layer kernel. When the number of convolution layers is lower, only the edge features of the image can be extracted. The more convolution layers, the more complex features can be iterated. However, as the convolution layer increases to a certain number, the training accuracy will stagnate or decline. Due to the increase of convolution layer, successive multi-layer gradient multiplication may cause minimum amount of information that is captured according to the chain rule in back-propagation, resulting in the problems of Gradient Vanishing^30^ and Gradient Exploding^31^ and the decline of the accuracy of the training set. Since spectrogram reflects 3-dimensional information through 2-dimensional images, more convolutional layers are needed to extract image features to iterate the depth feature information. Deep residual network (ResNet)^32^ model can be used to solve the problems of gradient vanishing and gradient exploding. ResNet model skips this layer or multi-layer operations through identity mapping, and directly transfers the gradient of the next layer to the upper layer in the back propagation process, which can improve the accuracy of the network with the increase of network layers.

**Figure 3:**
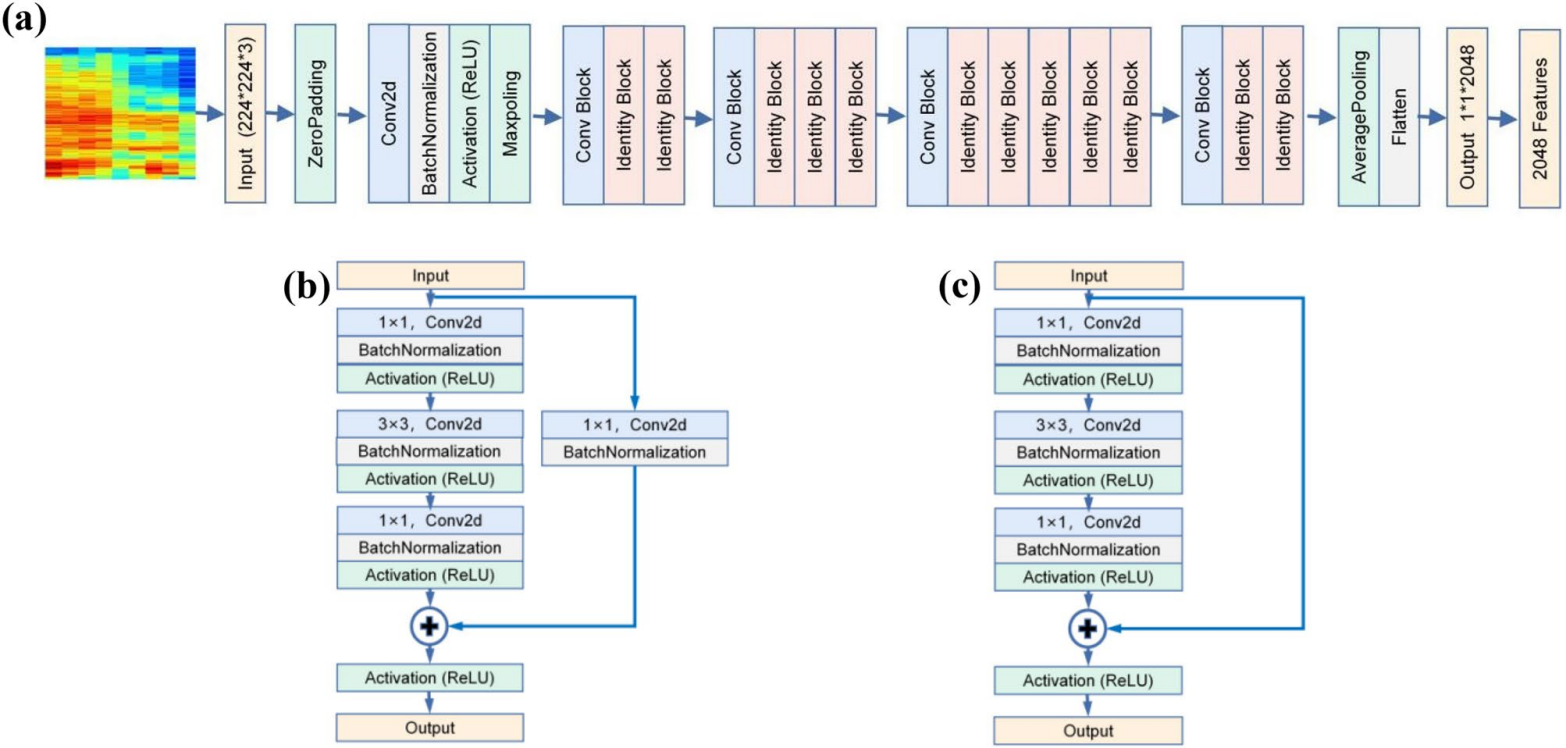
ResNet50 model feature extraction process. (**a**): Structure diagram of ResNet50 feature extraction model, in which 224*224*3 spectrogram is input and 2048-dimensional image features are output. (**b**): Detailed structure of ″Conv Block″ model in (**a**) to change the dimension of the network. (**c**): Detailed structure of ″identity Block″ module in (**a**) to deepen the function of feature extraction of the network.

Figure 3 presents the extraction process from cough spectrogram using ResNet50 mode. The full connection layer of the original ResNet50 model was removed and the flatten layer was set as the output after the average pooling layer. Figure 3b is the specific structure of ″Conv block″, which is used to change the features dimension; and Fig. 3c is the specific structure of the ″Identity block″, which is used to increase the ability of feature extraction of the model. Such skip connection allows the gradient to pass through this shortcut, which can alleviate the problem of gradient vanishing and gradient exploding, and ensure the stability of the model learning process.

#### Feature fusion

The subject's age, weight, height, and gender are used in the polynomial equation for calculating the pulmonary function parameter as reference values^33^. The biological attributes of the subjects refer to the basic physiological characteristics of the subject, such as age, sex, height, weight, etc. When patients with respiratory diseases have pulmonary function tests, they need to determine the normal value range of lung function parameters through information such as age, sex, height, weight and body surface area. Therefore, the biological attribute characteristics of the subjects were recorded to enhance the generalization and variability of the overall feature parameters.

In order to improve the accuracy of model evaluation, 2048-dimensional spectrogram features extracted through ResNet50 model fuse with the 4-dimensional biological attribute features to obtain the 2052-dimensional features. By comparing the evaluation indexes before and after the addition of biological attributes through ResNet50 + SVR hybrid model, it is tested to confirm the feature of biological attributes is conductive to estimate of pulmonary function parameters.

#### Feature normalization

In order to eliminate the effect of scale differences between features, the features are normalized. Formula (2) scales the values in cough feature to the average of 0 and a variance of 1, which can improve the model's accuracy.

2 $${\text{X}}({\text{i}})=\frac{{\text{X}}({\text{i}})-\overline{{\text{X}}}}{\upsigma }$$

where σ is the standard deviation of a single feature, and $\overline{{\text{X}} }$ is the mean value of a single feature.

### Support vector regression model

Support vector machine (SVM) is a small sample machine learning method with solid theoretical foundation^34^, which is appropriate to evaluate pulmonary function parameters with sample size of 371 in this work. Support vector regression (SVR) model uses hyperplane as the decision boundary to find the optimal support vector and to build the segmentation plane. In order to realize linearly separation, kernel function is applied to map the linear inseparable data to a higher-level high-dimensional feature space^35^. The final decision only needs a small number of support vectors, and is not restricted by the number of samples. After using the ResNet50 model for feature extraction, the 2048 dimensional features of each cough sound are fused with the corresponding biological attributes of the subjects to form a merged 2052 dimensional feature, which is input into the SVR model to evaluate pulmonary function parameters. Gray wolf optimization algorithm (GWO)^36^ is used to automatically optimize and adjust penalty factor C and kernel coefficient gamma in SVR model, and the RBF Gaussian kernel function is used to map the kernel function to the high-dimensional feature space. The parameters set by the model in this article are: ResNet50 Model parameter setting: Convolutional Kernel 7 × 7, Input_ Size = 224 × 224 × 3, Batch_ Size = 48, Epoch: 2000, Learning_ Rate = 1e-4, Optimizer = Adam, Activation Function is Relu. SVR model parameter settings: Kernel = RBF, C = 10. RBF kernel function is expressed by Formula (3):

3 $$R(x,c)=exp(-\frac{1}{2{\sigma }^{2}}{\Vert x-c\Vert }^{2})$$

where ${\text{x}}$ is the observed value, ${\text{c}}$ is the designated center point, and ${\upsigma }^{2}$ is the action range of the kernel function.

### Ethical approval

The protocol of this study has been approved by the ethical committee of Lishui People's Hospital, Zhejiang, China. Project reference: 2022(020-01).

## Results

In this work, four models are used for estimation and comparison, namely ResNet50 + SVR hybrid model, ResNet50 model, VGG16 + SVR hybrid model, and ResNet50 + RF hybrid model. The spectrogram of cough sounds is adjusted to the size of 224*224*3 as the model input, and the five pulmonary function parameters as the model output. Root Mean square error (RMSE), mean absolute error (MAE), and coefficient of determination (${{\text{R}}}^{2}$) are three evaluation indicators to evaluate the accuracy of the model. And fivefold cross-validation procedure to evaluate the performance and generalization ability of the model, to prevent overfitting. It randomly divides the training dataset into five subsets, each of which is called a 'fold'. One fold is selected for testing and the remaining four folds are used for training, and the performance of the model is evaluated using the test fold. This step will be repeated five times, and each fold will be tested once. After completing five iterations, take the average performance indicator RMSE、MAE and R^2^ of these five tests as the final performance evaluation of the model.

4 $${\text{RMSE}}=\sqrt{\frac{1}{{\text{m}}}{\sum_{{\text{i}}=1}^{{\text{m}}}({\text{yi}}-\ddot{{\text{yi}}})}^{2}}$$

5 $${\text{MAE}}=\frac{1}{{\text{m}}}\sum_{{\text{i}}=1}^{{\text{m}}}|{\text{yi}}-\ddot{{\text{yi}}}|$$

6 $${\text{R}}^{2} = 1 - {{\sum\limits_{{\text{i}}} {(\mathop {{\text{yi}}}\limits^{{..}} - {\text{yi}})} ^{2} } \mathord{\left/ {\vphantom {{\sum\limits_{{\text{i}}} {(\mathop {{\text{yi}}}\limits^{{..}} - {\text{yi}})} ^{2} } {\sum\limits_{{\text{i}}} {(\overline{{{\text{yi}}}} - {\text{yi}})} ^{2} }}} \right. \kern-\nulldelimiterspace} {\sum\limits_{{\text{i}}} {(\overline{{{\text{yi}}}} - {\text{yi}})} ^{2} }}$$

where ${\text{yi}}$ represents the model estimated value, $\ddot{{\text{yi}}}$ represents the real value, $\overline{{\text{yi}} }$ represents the mean of real value, and ${\text{m}}$ represents the number of samples.

### Experimental result

**Figure 4:**
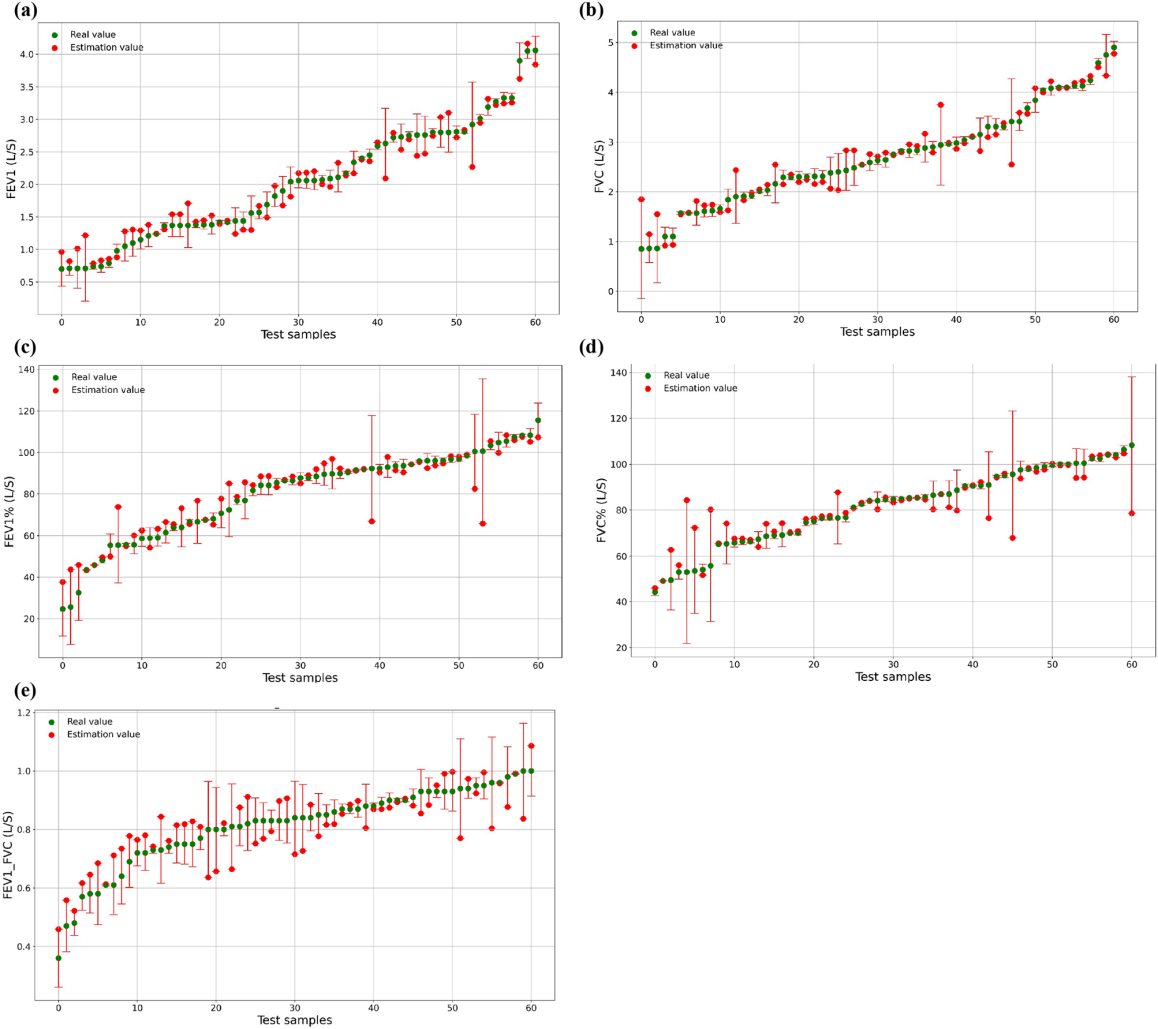
The best model estimation results of (**a**) FEV1, (**b**) FVC, (**c**) FEV1%, (**d**) FVC%, and (**e**) FEV1/FVC based on ResNet50 + SVR hybrid model.

Table 2 presents the evaluation indicators for estimating five pulmonary function parameters, and ResNet50, ResNet18, and VGG16 models are used for comparison. It is clear that ResNet50 + SVR hybrid model can obtain lower RMSE and MAE values than other models. For the estimation of FEV1 and FVC, the ResNet50 + SVR hybrid model achieves the best accuracy. It is also noted that the ResNet50 + SVR hybrid model shows the best performance in FEV1/FVC, FEV1%, and FVC%. The comparison between the model estimated values and the real values of the five pulmonary function parameters using the ResNet50 + SVR hybrid model is shown in Fig. 4.

1. For evaluating the FEV 1 parameters, the ResNet50 + SVR model achieved the best accuracy, with RMSE = 0.2, MAE = 0.16 and = 0.95, respectively.
2. For evaluating the FVC parameters, the ResNet50 + SVR model achieved the best accuracy, with RMSE = 0.28, MAE = 0.19 and = 0.92, respectively.
3. For evaluating the FEV1/FVC parameters, the ResNet50 + SVR model achieved the best accuracy, with RMSE = 0.08, MAE = 0.06 and = 0.68, respectively.
4. For evaluating the FEV 1% parameter, the ResNet50 + SVR model achieved the best accuracy, with RMSE = 8.25, MAE = 5.1 and = 0.85, respectively.
5. For evaluating the FVC% parameters, the ResNet50 + SVR model achieved the best accuracy, with RMSE = 8.62, MAE = 4.5 and = 0.73, respectively.

**Table 2:** Comparison of the performances of multiple models.

Model	Indicators	FEV1	FVC	FEV1/FVC	FEV1%	FVC%
ResNet50 + SVR	RMSE	0.20	0.28	0.08	8.25	8.62
	MAE	0.16	0.19	0.06	5.10	4.50
	R^2^	0.95	0.92	0.68	0.85	0.73
ResNet50	RMSE	1.05	1.13	0.15	25.89	22.70
	MAE	0.85	0.91	0.11	21.23	17.83
	R^2^	− 0.27	− 0.44	− 0.41	− 0.35	− 0.52
ResNet18 + SVR	RMSE	0.25	0.34	0.11	8.45	10.69
	MAE	0.17	0.26	0.09	5.24	7.43
	R^2^	0.92	0.87	0.27	0.82	0.57
ResNet18	RMSE	1.02	1.09	0.15	22.64	20.66
	MAE	0.86	0.90	0.12	17.67	14.99
	R^2^	− 0.28	− 0.24	− 0.27	− 0.05	− 0.41
VGG16 + SVR	RMSE	0.58	0.49	0.16	24.90	17.91
	MAE	0.46	0.39	0.12	20.02	14.34
	R^2^	0.61	0.74	0.06	0.01	0.05
VGG16	RMSE	1.06	1.09	0.15	24.07	17.55
	MAE	0.86	0.90	0.13	20.27	13.75
	R^2^	− 0.04	− 0.18	− 0.09	− 0.05	− 0.21

### Effects of difference regression models

In order to verify the impact of different regression models on the evaluation of pulmonary function parameters, ResNet50 was used to extract 2048-dimensional features and 4-dimensional biological attribute features. Three regression models with better performance were used for comparison, and the evaluation results of different regression models were compared in Table 3. It is seen that using support vector regression model to evaluate pulmonary function parameters can significantly reduce RMSE and MAE, and greatly improve R^2^. The improvement rate of each parameter is FEV1 (38.2%), FEV1% (18.3%), FVC (41.5%), FVC% (18%), and FEV1/FVC (11.5%), respectively.

**Table 3:** Comparison of the performances of difference regression models.

Model	Indicators	FEV1	FVC	FEV1/FVC	FEV1%	FVC%
ResNet50 + SVR	RMSE	0.20	0.28	0.08	8.25	8.62
	MAE	0.16	0.19	0.06	5.10	4.50
	R^2^	0.95	0.92	0.68	0.85	0.73
ResNet50 + GBR	RMSE	0.60	0.56	0.20	19.78	17.90
	MAE	0.41	0.41	0.15	15.09	13.17
	R^2^	0.59	0.67	− 0.56	0.38	0.05
ResNet50 + RF	RMSE	0.54	0.54	0.14	17.77	14.88
	MAE	0.36	0.38	0.11	14.27	11.17
	R^2^	0.67	0.69	0.19	0.50	0.34

### Effects of biological attribute features

Table 4 shows the effect of biological attribute features on the accuracy of regression models. By adding biological attributes into ResNet50 + SVR model, RMSE and MAE were significantly reduced. It is shown that the introduction of biological attributes leads to a significant improvement in ${{\text{R}}}^{2}$. Significantly, biological attribute features have a greater impact on directly measured parameters, and less impact on proportional parameters.

**Table 4:** Compare performance before and after adding biological attributes based on ResNet50 + SVR.

Model	Indicators	FEV1	FVC	FEV1/FVC	FEV1%	FVC%
Model(SVR) with sex, age, weight and height	RMSE	0.20	0.27	0.07	8.70	9.41
	MAE	0.14	0.19	0.05	4.35	3.78
	${{\text{R}}}^{2}$	0.94	0.92	0.68	0.84	0.72
Model(SVR) without sex, age, weight and height	RMSE	0.53	0.58	0.10	13.44	9.88
	MAE	0.23	0.25	0.07	6.85	4.01
	${{\text{R}}}^{2}$	0.68	0.65	0.61	0.71	0.61

### Effect of homologous cough sounds

The previous experiment used 371 coughs from 150 patients to evaluate pulmonary function, but homologous cough sounds can easily affect the model's judgment of unknown cough sounds. Therefore, in order to verify the impact of homologous cough sounds, we collected independent cough sounds from 650 clinical subjects for evaluation. The 650 subjects only coughed once per person, producing a total of 650 cough sounds. The dataset were divided the test and training sets an 8:2 ratio. Table 5 shows the results of the evaluation. The results showed that multiple cough sounds from subjects with the same disease, especially when the total amount of data is not particularly large, did indeed have an impact on lung function parameters, especially on the performance of FEV1 and FVC parameters. However, there was an increase in performance for FEV1%, FVC%, and FEV1/FVC parameters, indicating that increasing data volume and ensuring data independence are very helpful in improving parameter performance.

**Table 5:** Pulmonary function evaluation experiment based on independent cough sounds of 650 subjects.

Model	Indicators	FEV1	FVC	FEV1/FVC	FEV1%	FVC%
Independent cough sounds validation model for 650 subjects(ResNet50 + SVR)	RMSE	0.33	0.33	0.06	8.24	7.35
	MAE	0.19	0.18	0.04	4.01	3.11
	${{\text{R}}}^{2}$	0.84	0.87	0.73	0.85	0.82

## Conclusion

Cough sound carries a variety of information on pulmonary health condition. In this study, the deep learning model (i.e., ResNet50) and machine learning model (i.e., SVR) are combined to realize the process of cough sounds estimation for pulmonary function parameters. Firstly, the cough sounds are converted into spectrogram images using STFT. Secondly, the spectrogram is input into the ResNet50 model for extracting 2048-dimensional features from images. Finally, 2048-dimensional features are fused with 4-dimensional human biological attributes after standardization, and input into the SVR regression model to estimate the pulmonary function parameters. The experimental results show that a high estimating effect can be achieved through this method. The R^2^ of each estimating pulmonary function parameters is 0.94, 0.92, 0.68, 0.84, and 0.72 for FEV1, FVC, FEV1/FVC, FEV1%, and FVC%, respectively.

Pulmonary function testing is the gold standard for evaluating pulmonary function, but it cannot meet the daily monitoring needs of patients with lung diseases. It is normal for problems to occur during the examination process. The process of coughing has some similar to pulmonary function testing, and the cough sound contains rich respiratory information, which can be achieved easily. For cough sounds and pulmonary function parameters, we evaluate cough sounds by analyzing the relationship between cough sound and pulmonary function parameters. It is expected to gain its new clinical and disease control value in the diagnosis of respiratory diseases, so as to facilitate the daily lung function examination of patients with chronic respiratory diseases, and reduce the delay and aggravation of patients caused by not timely diagnosis.This technology has great potential, and in the future, it can provide practical basis and reliable technology for large-scale screening of possible unknown infectious diseases. As a technical accumulation, this study can provide a large-scale screening means for major respiratory diseases that may occur in the future, and reduce the loss of personnel and especially medical workers and the economic burden of government. However, this paper only provides a preliminary discussion, there must be shortcomings, need to be further improved. Such as the small sample size of 150 cases, which cannot represent a larger population. In addition, there is also sampling bias, with the majority of the samples being adults and elderly people, lacking representation. Rudraraju's research^37^ also uses cough sounds as input signals, which shares many similarities with the paper. They attempted to provide detailed disease classification results, while we only attempted to evaluate the main lung function parameters. Of course, the results will serve as a diagnostic basis. It is worth emphasizing that this study is only used as an auxiliary method for medical diagnosis and evaluation. Due to the complexity of the disease, the diagnosis process can not be limited to this technique. When a cough or discomfort or the type and nature of the disease need to be determined, we also need to go to the hospital for a series of multi-system examinations. The method verifies its effectiveness with only a small amount of data, and can not meet the requirements of clinical practice in terms of accuracy and universality, and still has a long way to go before practical application.

## Data Availability

The datasets generated and analyzed during the current study are not publicly available due permission restrictions, but are available from the corresponding author on reasonable request.
